# Treatment options in platinum-refractory male germ cell cancers: current standards and future directions

**DOI:** 10.1177/17588359261451841

**Published:** 2026-05-31

**Authors:** Marco Oltrecolli, Domiziana Alaimo, Fabrizio Di Costanzo, Giovanni Rosti, Pasquale Rescigno, Massimo Dominici, Winfried Alsdorf, Carsten Bokemeyer, Christoph Oing, Christoph Seidel

**Affiliations:** Northern Centre for Cancer Care, Freeman Hospital, The Newcastle upon Tyne Hospitals NHS Foundation Trust, Newcastle upon Tyne, UK; Department of Oncology and Hematology, Azienda Ospedaliero-Universitaria of Modena, Modena, Italy; Northern Centre for Cancer Care, Freeman Hospital, The Newcastle upon Tyne Hospitals NHS Foundation Trust, Newcastle upon Tyne, UK; Department of Internal Medicine and Medical Therapy, University of Pavia, Pavia, Italy; Department of Oncology, Comprehensive Cancer Centre, Fondazione IRCCS Policlinico San Matteo, Pavia, Italy; Northern Centre for Cancer Care, Freeman Hospital, The Newcastle upon Tyne Hospitals NHS Foundation Trust, Newcastle upon Tyne, UK; Department of Medical Oncology, Università degli Studi di Napoli Federico II, Naples, Italy; Department of Oncology, Comprehensive Cancer Centre, Fondazione IRCCS Policlinico San Matteo, Pavia, Italy; Northern Centre for Cancer Care, Freeman Hospital, The Newcastle upon Tyne Hospitals NHS Foundation Trust, Newcastle upon Tyne, UK; Translational and Clinical Research Institute, Newcastle University, Newcastle upon Tyne, UK; Department of Oncology and Hematology, Azienda Ospedaliero-Universitaria of Modena, Modena, Italy; Department of Oncology, Hematology and Stem Cell Transplantation with Division of Pneumology, University Medical Centre Hamburg-Eppendorf, Hamburg, Germany; Department of Oncology, Hematology and Stem Cell Transplantation with Division of Pneumology, University Medical Centre Hamburg-Eppendorf, Hamburg, Germany; Northern Centre for Cancer Care, Freeman Hospital, The Newcastle upon Tyne Hospitals NHS Foundation Trust, Newcastle upon Tyne, UK; Translational and Clinical Research Institute, Newcastle University, Newcastle upon Tyne, UK; Department of Oncology, Hematology and Stem Cell Transplantation with Division of Pneumology, University Medical Centre Hamburg-Eppendorf, Hamburg, Germany; Department of Oncology, Hematology and Stem Cell Transplantation with Division of Pneumology, University Medical Centre Hamburg-Eppendorf, Hamburg 20246, Germany

**Keywords:** antibody–drug conjugates, cellular therapy, chemotherapy, cisplatin resistance, germ cell cancer, testicular cancer

## Abstract

Germ cell cancers (GCCs) represent the most common malignant tumours among men up to the age of 40 years. GCCs are characterised by high cure rates of >99% for localised stage I disease, but even at advanced metastatic stages, 67%–96% of patients can be cured with multimodal treatment approaches. The high curability owes to an exceptional responsiveness to cisplatin-based chemotherapy. However, about 30% of patients with metastatic disease eventually relapse after first-line multimodality treatment. At first relapse, salvage treatment still cures approximately 50% of patients. Platinum-based chemotherapy remains the standard of care in this setting, administered either as conventional-dose cisplatin-based regimens or as high-dose carboplatin-based combination chemotherapy. Across multiple large retrospective cohorts, high-dose chemotherapy has repeatedly been associated with superior progression-free and overall survival compared with conventional-dose approaches, although definitive prospective evidence is awaited. Patients suffering second or multiple relapses are considered platinum-resistant and face a very dismal prognosis with a life expectancy generally limited to less than 12 months. The mainstay of treatment in this setting is still conventional cytotoxic chemotherapy since no molecularly targeted treatments have shown clinically meaningful activity, to date. However, Claudin-6 (CLDN6)-directed approaches appear the most promising avenue of new treatment options in this situation. In this narrative review, we outline established and emerging treatment opportunities for this rare but challenging clinical scenario of platinum-resistant GCCs. This review provides a focused and up-to-date synthesis of therapeutic options for multiply relapsed, platinum-refractory GCCs, with a particular emphasis on late-line treatment sequencing and emerging biomarker-driven strategies, including CLDN6-directed therapies.

## Plain language summary

New treatment options for germ cell cancer when standard chemotherapy no longer works

Germ cell cancers (GCCs) are highly curable tumours, even when they have spread. Most patients respond very well to standard cisplatin-based chemotherapy. However, a small group of patients experience several relapses and eventually develop resistance to platinum-based treatment. In this situation, treatment options are limited and long-term survival is rare. This review summarises the available therapies for patients with platinum-refractory GCCs. High-dose chemotherapy can still help some patients, but its success decreases after multiple relapses. Standard combination chemotherapies may temporarily shrink the tumour, but responses usually do not last. Modern treatments such as targeted drugs or immunotherapy have shown very limited benefit to date. A promising new approach targets a protein called Claudin-6 (CLDN6), using innovative treatments such as antibody–drug conjugates or CAR-T cells. These early results offer hope for future therapeutic progress.

## Background

GCCs are relatively rare, representing about 1% of all cancers in men; however, they are the most common cancer among young men aged 15–40.^1^ The incidence rate varies globally, with higher rates in Western countries and lower rates in Asia and Africa. While the overall incidence is increasing, survival rates are high even in metastatic stages with 5-year survival rates from 67% to 96% according to the International Germ Cell Cancer Collaborative Group-Update Consortium for patients with advanced disease due to excellent response to platinum-based chemotherapy.^2^

Despite the generally outstanding chemotherapy sensitivity, 20%–30% of patients relapse after or progress during cisplatin-based first-line treatment and require salvage treatment with either conventional-dose chemotherapy (CDCT) or high-dose chemotherapy (HDCT) plus autologous stem cell transplant (ASCT).^3^ Across the literature, the terms platinum-refractory and platinum-resistant are not used uniformly, which can limit cross-trial comparability – particularly when translational cohorts are included. In this review, we use platinum-refractory disease to denote progression during cisplatin-based chemotherapy or relapse within a short interval after completion of a cisplatin-containing regimen. By contrast, platinum-resistant disease refers to relapse after an initial response to cisplatin-based therapy, typically occurring beyond this early interval. We explicitly acknowledge that individual clinical trials and translational studies apply heterogeneous time cut-offs and response requirements, which needs to be considered when interpreting reported outcomes.^3^

Salvage chemotherapy in combination with post-chemotherapy residual tumour resection can achieve longer lasting remissions and eventually cure in approximately 50% of relapsed patients.^4^ Subsequent relapses are associated with substantially worse outcomes. Overall, 3%–5% of all GCCs patients develop platinum-refractory disease and potentially die from GCC.^5^ Unfortunately, treatment options after failure of two or more lines of platinum-based combination treatments are limited and patient survival is generally limited to a few months only.^6^ Despite the widespread use of targeted and molecular therapies in other cancers, their development in GCCs has shown little success to date. In this review, we aim to explore current and emerging therapeutic strategies for multiply relapsed, platinum-refractory GCC patients.

## HCDT for multiply relapsed GCC patients

High-dose salvage chemotherapy with two or three cycles of a doublet regimen consisting of high-dose carboplatin and etoposide followed by ASCT is typically considered as first salvage treatment. Retrospective registry data suggest that HDCT may still achieve cure in multiply relapsed GCC patients if not previously administered. However, durable responses are less frequent when HDCT is used as a third or later line of treatment compared to its use as first or second salvage therapy following failure of a CDCT. According to a retrospective analysis from Indiana University, the 2-year progression-free survival (PFS) rate among 61 patients treated with HDCT as third or subsequent line of therapy was still 50%.^7^ Seidel et al. conducted a retrospective observational cohort study including 283 patients treated at 11 international institutions. All patients had relapsed or refractory GCC of which 124 patients had undergone salvage HDCT after at least one salvage treatment line. The median follow-up for these patients was 17.0 months, and among these patients, the objective response rate (ORR) was 60% and the 2-year and 5-year overall survival (OS) rates were 53% and 37%, respectively. Importantly, HDCT as third or further line treatment was associated with a higher incidence of grade ⩾3 non-haematologic toxicities than when used as first salvage treatment.^8^

In the retrospective analysis by Lorch et al.,^9^ HDCT as single or sequential therapy yielded responses in 55% of patients but resulted in a disappointing overall prognosis, with a projected 5-year OS of only 17%.

While HDCT remains an important curative option, it is associated with substantial acute and long-term toxicity. Acute complications include severe myelosuppression with febrile neutropenia and invasive infections, mucositis, gastrointestinal toxicity and the need for intensive supportive care, including transfusions and prolonged hospitalisation. Treatment-related mortality, although reduced in experienced centres, remains a relevant risk in heavily pretreated patients. In addition, cumulative non-haematologic toxicities may occur after multiple prior lines, including nephrotoxicity, ototoxicity, neurotoxicity, pulmonary toxicity and impaired marrow reserve. Long-term survivorship issues – particularly relevant in this young population – include infertility, endocrine dysfunction and the potential risk of secondary malignancies after extensive cytotoxic exposure. These risks underscore the importance of careful patient selection, treatment in expert centres and early integration of survivorship counselling and fertility preservation.^7,10^ In conclusion, HDCT in combination with post-chemotherapy resection of residual tumours can still be pursued with curative intent even when used as second or subsequent salvage treatment. However, reported long-term survival rates in this setting vary considerably across the literature and are generally markedly lower than those observed when HDCT is administered as first salvage therapy.

Durable disease control in these late-line settings is most consistently reported in patients who undergo complete post-chemotherapy resection of all residual disease whenever feasible. However, study-level reporting stratified by resection status and detailed relapse histology is inconsistent across cohorts, limiting more granular comparisons.

## Palliative chemotherapy treatments

Due to their young age and general lack of relevant co-morbidities, refractory GCC patients are often capable of tolerating third and further line combination chemotherapy, even after HDCT with autologous stem cell transplantation.^6^ Table 1 highlights available evidence for active single agent and combination chemotherapy regimens in this setting.

**Table 1.** Chemotherapy regimens for palliative setting.

Agent(s)	Schedule	ORR	Duration of benefit for responders (measure, median, range; months)	Prior HDCT	Reference
Oxaliplatin	60 mg/m^2^ iv d1,8,15 q3w	6%	PFS, n.r. (2–8) 78%		(11)
	130 mg/m^2^ iv d1 q2w	19%			
	130 mg/m^2^ iv d1 q3w	25%	PFS, n.r.	NR	(12)
Gemcitabine	1000 mg/m^2^ iv d1,8,15 q3w	19%	TTF, 4+ (2–9+)	71%	(13)
	1200 mg/m^2^ iv d1,8,15 q3w	15%	PFS, n.r. (2–6)	55%	(14)
Paclitaxel	135–310 mg/m^2^ iv d1 q3w	20%	DOR, n.r. (3–5)	NR	(15)
	170 mg/m^2^ iv d1 q3w	11%	PFS, n.r. (1.5–2)	16%	(16)
	225 mg/m^2^ iv d1 q3w	25%	DOR, 8+ (3–16+)	50%	(17)
	250 mg/m^2^ iv d1 q3w	13%	DOR, n.r. (9–10 weeks)	13%	(18)
	250 mg/m^2^ iv d1 q3w	26%	n.r.	16%	(19)
Oral etoposide	50 mg/m^2^ po q3w	14%	n.r.	29%	(20)
Temozolomide	150–200 mg/m^2^ po d1–5 q4w	10%	DOR, 1.5 (3.5–9)	40%	(21)
GEMOX	Gemcitabine 1000 mg/m^2^ iv d1,8 q3w	46%	PFS, n.r. (2–16+)	89%	(22)
	Oxaliplatin 130 mg/ m^2^ iv d1 q3w				
	Gemcitabine 1000 mg/m^2^ iv d1,8 q3w	32%	PFS. n.r. (2–28+)	14%	(23)
	Oxaliplatin 130 mg/m^2^ iv d1 q3w				
	Gemcitabine 1250 mg/m^2^ iv d1,8 q3w	17%	PFS, n.r. (18+–44+)	22%	(24)
	Oxaliplatin 130 mg/m^2^ iv d1 q3w				
OXIRI	Oxaliplatin 85 mg/m^2^ iv d1,15 q4w	39%	PFS, n.r. (2–19+)	0	(25)
	Irinotecan 80 mg/m^2^ iv d1,8,15 q4w				
GOP	Gemcitabine 800 mg/m^2^ iv d1,8 q3w	51%	PFS, 3 (1–17+)	78%	(26)
	Oxaliplatin 130 mg/m^2^ iv d1 q3w				
	Paclitaxel 80 mg/m^2^ iv d1,8 q3w				
	Gemcitabine 800 mg/m^2^ iv d1,8 q2w	31%	PFS, 6.5 (95% CI, 3.2–27.5)	20%	(27)
	Oxaliplatin 100–125 mg/m^2^ iv d1 q2w				
	Paclitaxel 170 mg/m^2^ iv d1 q2w				
TPG	Paclitaxel 80 mg/m^2^ iv d1,8 q3w	49.4%	2-year PFS 14.8 % (95% CI, 8.5%–25.8 %)	13.3%	(28)
	Cisplatin 50 mg/m^2^ iv d1,8 q3w				
	Gemcitabine 800 mg/m^2^ iv d1,8 q3w				
EMA/CO	Etoposide 100 mg/m^2^ iv d1,2 q2w	29%	PFS, 3 (95% CI, 2–4)	39%	(29)
	Methotrexate 300 mg/ m^2^ iv d1 q2w				
	Folinic acid 15 mg × 4 iv/po d2,3 q2w				
	Actinomycin D 0.5 mg/m^2^ iv d1,2 q2w				
	Vincristine 1 mg/m^2^ iv d8 q2w				
	Cyclophosphamide 600 mg/m^2^ iv d8 q2w				

CI, confidence interval; DOR, duration of response; EMA CO, etoposide/methotrexate/actinomycin D alternating with cyclophosphamide/vincristin; GEMOX, gemcitabine/oxaliplatin; GOP, gemcitabine/oxaliplatin/paclitaxel; HDCT, high-dose chemotherapy; n.r./NR, not reported; ORR, objective response rate; OXIRI, oxaliplatin/irinotecan; PFS, progression-free survival; TPG, paclitaxel/cisplatin/gemcitabine; TTF, time to treatment failure.

### Monochemotherapy

As outlined above, cytotoxic chemotherapy remains the backbone of treatment for refractory GCCs. Several single-arm phase II studies have historically evaluated single-agent cytotoxic drugs in the platinum-resistant setting – including oral etoposide, paclitaxel, gemcitabine, oxaliplatin and ifosfamide – typically reporting ORRs of only 10%–20% and durable remissions in merely 3%–10% of patients.^5^

More recently, cabazitaxel has been explored as a modern late-line option in small phase II and compassionate-use cohorts, typically after failure of multi-agent regimens such as GOP.^30^ While cabazitaxel shows activity in individual patients, responses remain infrequent and rarely durable, underscoring the biological refractoriness of this disease stage.

Given the overall modest activity and lack of sustained disease control, single-agent chemotherapy is generally reserved for patients who are not fit for combination regimens owing to poor performance status, comorbidities or cumulative toxicities affecting marrow or renal reserve.^31^

### Polychemotherapy

#### Doublet regimens

Based on active single agents, a variety of combination chemotherapy regimens have been established and adopted in the management of platinum-refractory GCC patients.

##### Gemcitabine and oxaliplatin

The doublet combination of gemcitabine and oxaliplatin (GEMOX) is a well-established treatment regimen in the palliative setting, and it was assessed in two-phase II trials.^22,23^ In the first study, GEMOX was administered to 35 GCC patients, of which approximately half had previously undergone HDCT. Sixteen pts (46%) achieved a response, with 3 (9%) achieving complete remission, 2 following chemotherapies alone and 1 after post-chemotherapy surgical resection.^22^

##### Oxaliplatin and irinotecan

Two trials evaluated the doublet combination of oxaliplatin and irinotecan (OXIRI), administered alone or in combination with paclitaxel, showing that the addition of paclitaxel resulted in a higher ORR (70% vs 40%). Nevertheless, these studies excluded patients who had previously undergone HDCT, which may overestimate activity of these combinations in patients who had prior salvage HDCT.^25,32^

#### Triplet regimens

The highest response rates so far have been achieved with two triplet combination chemotherapy regimens demonstrating ORRs of more than 50%, in patients who did not receive paclitaxel before: gemcitabine, oxaliplatin and paclitaxel (GOP) and paclitaxel, gemcitabine and cisplatin (TPG).^26,28^ Importantly, long-term disease control can generally only be achieved in subjects who undergo post-chemotherapy surgical removal of all residual masses.^33^ Of note, the available evidence rarely provides uniform, granular outcomes stratified by completeness of resection (complete vs incomplete vs no surgery) or by detailed viable relapse histology (e.g. necrosis/teratoma/viable non-teratoma, somatic malignant transformation), which represents a key limitation of the current literature.

##### Gemcitabine, oxaliplatin and paclitaxel

Two phase II trials and a retrospective cohort study confirmed the efficacy of the triplet combination of GOP.^26,27,34^ Firstly, a phase II German Testicular Cancer Study Group (GTCSG) trial enrolled 41 patients with treatment-refractory disease, where approximately 80% of them had failed previous HDCT. These patients received a 3-weekly GOP regimen yielding an ORR of 51%. Two patients achieved a complete response (CR) with chemotherapy alone, while six others had no evidence of disease (NED) after post-chemotherapy surgical resection.^26^ Another phase II trial reported similar treatment efficacy using a bi-weekly application schedule. Specifically, an ORR of 31% was observed with 17% of patients reaching CR after surgery and 7% a CR following GOP alone.^27^ Finally, the GTCSG conducted a retrospective cohort study with 63 patients treated with the 3-weekly GOP regimen, demonstrating an ORR 44% and median OS and PFS of patients who achieved ORR were 22.6 months (95% CI: 0.00–48.47) and 10.2 months (95% CI: 4.31–16.01), respectively.^34^

##### Gemcitabine, cisplatin and paclitaxel

Another triplet regimen, TPG (gemcitabine, cisplatin and paclitaxel), demonstrated a nearly 50% ORR in a cohort of 75 patients who had progressed after two or three cisplatin-based therapy schedules, comprising HDCT. Notably, more than half of these patients were cisplatin-refractory. A partial response (PR) with normalised tumour markers (PRm-) was observed in 29 subjects (38.7%), while eight patients (10.7%) obtained a CR, with median OS and PFS being 13 and 5 months.^28^

#### Methotrexate/etoposide/actinomycin D administered in sequence with cyclophosphamide and vincristine

Concerning patients with hCG positive germ cell cancers (GCCs), in particular those with elements of chorion carcinoma, the methotrexate/etoposide/actinomycin D administered in sequence with cyclophosphamide and vincristine (EMA/CO) regimen displays another treatment option. Developed in the 1980s, it was designed for the treatment of high-risk gestational trophoblastic neoplasia, particularly in patients resistant to single-agent methotrexate or actinomycin D. The rationale was to combine agents with different mechanisms of action and non-overlapping toxicities in a dose-dense, alternating schedule to maximise efficacy while maintaining tolerability. The regimen emerged after earlier experiences with the EMA triplet (etoposide, methotrexate, actinomycin D) demonstrated high activity. Modifications such as EMA/EP (etoposide and cisplatin replacing CO) have been developed for resistant cases or as salvage options. In a retrospective registry with 41 treatment-resistant hCG-positive patients, who received this combination regimen, now established as an option for this setting, just under one-third of patients reached objective responses and 12% attaining a CR. OS and PFS were 8 and 3 months, respectively.^29^ This limited activity may be due to heavy pre-treatment in this patient population, and further real-world data ares needed to better understand the role of EMA/CO and EMA/EP for this patient population.

## Small molecule inhibitors

### Tyrosine kinase inhibitors

Receptor tyrosine kinases (RTKs) have been shown to be activated in pre-treated GCC patients. The RTKs most commonly involved include platelet-derived growth factor, vascular endothelial growth factor receptor, hepatocyte growth factor receptor, stem cell growth factor receptor and epidermal growth factor receptor. However, although extensively evaluated in multiple clinical trials, RTK-targeted therapies have yet failed to produce clinically meaningful responses in unselected platinum-resistant GCC patient cohorts.^31^

Importantly, most of these studies were conducted without prior comprehensive molecular profiling and molecular patient selection, and therefore largely tested tyrosine kinase inhibitors (TKIs) in unselected GCC patients, which may in fact have had detrimental effect on TKI activity in these trial populations.

Sunitinib is a multikinase inhibitor administered in 32 refractory germ cell tumour (GCT) patients enrolled in a phase II trial, showing a 13% of total response rate (only PR) with a median OS and median PFS of 3.8 months (95% CI, 3–6.6) and 2 months (95% CI, 1.4–2.6), respectively.^35^ Another phase II trial included 43 patients treated with pazopanib in monotherapy, another multi-kinase inhibitor, reported a tumour marker response in approximately 70% of cases. Transient radiographic disease stabilisation was achieved in 44%, but only 4.7% achieved a PR. After a median follow-up of 29.6 months (range = 10.6–35.8 months), the 24-month OS rate was 14.2% (95% CI, 6.0%–33.7%) and 3-month PFS rate was 12.8% (95% CI, 5.7%–28.9%).^36^ Sorafenib was evaluated in a phase II trial that enrolled 18 patients with refractory GCTs, reaching disease stabilisation lasting more than 1 year in 16.7% of cases (3 patients) and a reduction serum tumour marker in 44.4% of subjects (8 patients).^37^

Cabozantinib is a multikinase inhibitor targeting mainly mesenchymal-epithelial transition (MET) factor. It is currently being administered as monotherapy in an ongoing phase II trial involving refractory GCT patients, who relapsed after one salvage treatment. Notably, this trial is also investigating potential biomarkers, including next-generation sequencing and c-MET expression assessed by immunohistochemistry (IHC), with the aim of identifying predictors of response and resistance to therapy (NCT04876456).^38^ Between 10% and 40% of GCTs harbour c-KIT mutations, another RTK often overactivated in seminomas, but less frequently in non-seminomas. These mutations predominantly involve exon 17, which is commonly associated with resistance to c-KIT-targeted therapy. This might explain the limited clinical efficacy observed in a phase II trial evaluating imatinib, a predominant c-KIT inhibitor, in 18 refractory GCC patients, where no patient achieved an objective radiographic response and only one patient had a 3-months disease stabilisation.^39^ Across drug classes, the lack of systematic molecular profiling and biomarker enrichment is a recurring limitation in the late-line GCC trial landscape and should be addressed prospectively.

### mTOR inhibitors

An immunohistochemical study revealed a strong activation of mTOR pathway in seminomas, while loss of PTEN gene was also frequently observed in GCTs, prompting the evaluation of Everolimus, an mTOR inhibitor, in this setting. Two phase II trials were conducted, enrolling 15 and 25 patients. However, neither trial demonstrated any objective response, and the median OS and median PFS were less than 4 months and less than 2 months, respectively, in both studies.^40,41^

### Poly(ADP-ribose)polymerase inhibitors

One of the most important types of DNA damage caused by cisplatin is DNA single-strand breaks (SSB) and resulting replication-associated DNA double-strand breaks (DSB). Poly(ADP-ribose)polymerase (PARP) enzymes are key players of cellular SSB repair. PARP inhibitors (PARPi) disrupt SSB repair which causes replication-associated DSBs, which need to be repaired by homologous recombination (HR). In case of HR-deficiency, PARPi-induced DSB remain unrepaired which can lead to cell death.^42^ An in vitro study highlighted that the concomitant administration of PARPi and low-dose cisplatin in patients with cisplatin-refractory disease could be effective building on a PARPi-induced platinum-sensitisation in a synergistic manner.^43^ According to what was mentioned above, two separate trials investigated the efficacy of PARPi in heavily pretreated GCTs patients.

Olaparib was assessed in a phase II trial involving patients who had received at least two previous treatments. Among the 18 subjects enrolled, 5 patients achieved stable disease (SD; 27.8%), while no PRs were observed in the remaining participants. The 12-month OS was 27.8% (95% CI, 10.1%–48.9%), while 12-week PFS was also 27.8% (95% CI, 10.1%–48.9%). Germline DNA was analysed using target sequencing, and only one patient was identified as BRCA1 mutation carrier; this individual maintained SD over a period of 4 months.^44^ Another phase II study evaluated the PARPi veliparib in combination with carboplatin and gemcitabine. Fifteen patients were enrolled; SD or PR were documented in 5 (33.3%) and 4 (26.7%) cases, respectively. The primary aim of this study was 12-months PFS, but it was achieved only by one patient (6.7%). The median OS was 10.5 months (95% CI, 8.9–11.1), while the median PFS was 3.1 months (95% CI, 2.2–3.9).^45^ In conclusion, so far, PARPi fail to demonstrate meaningful therapeutic efficacy in a GCC setting. Possible reasons include cross-resistance between PARPi and platinum-based agents, inter- and intra-patient heterogeneity observed in refractory GCCs and the absence of predictive genetic alterations and biomarkers associated with PARPi responsiveness.^46^ The prevalence of somatic and germline homologous recombination repair (HRR) defects in GCTs is low.^44^ Notably, in cohort of 187 GCT patients, approximately 25 individuals exhibited at least one germline mutation in a DNA damage repair gene despite no reported familial predisposition to cancer, and around 5 of these cases involved BRCA1/2 mutations.^47^

## Epigenetic targeting

Aberrant DNA methylation of CpG islands within gene promotors silences gene expression and is mediated by DNA methyltransferases (DNMTs), which are frequently overexpressed in non-seminomatous GCCs.^48^ Consequently, non-seminomas display a globally hypermethylated genome, whereas seminomas are generally hypomethylated.^49^ Promoter hypermethylation of *RASSF1*, *HIC1*, *MGMT* and *CALCA* has been linked to non-seminomatous histology, and methylation of *RASSF1*, *HIC1* and *CALCA* correlated with reduced cisplatin sensitivity.^50,51^ In addition, a subset of non-seminomas shows hypermethylation of HRR genes (*BRCA1*, *RAD51C*), which was associated with PARPi sensitivity in preclinical models.^52^

Demethylating agents such as 5-azacytidine or 5-aza-deoxycytidine can reverse aberrant methylation and restore TP53-mediated apoptosis, particularly in *DNMT3B*-overexpressing embryonal carcinoma models.^53^ These agents also sensitise tumour cells to cisplatin.^48,54,55^ In a phase I trial, guadecitabine combined with cisplatin achieved a 23% response rate in heavily pretreated, platinum-refractory GCCs, with manageable haematologic toxicity.^56^ Ongoing trials are expected to define the therapeutic relevance of guadecitabine-based epigenetic modulation and to establish whether hypomethylating agents can be leveraged to achieve durable re-sensitisation to cisplatin in patients with refractory GCCs.

## Immunotherapy

Programmed cell death protein 1 (PD-1) is an inhibitory receptor expressed on multiple immune cells, including T cells, NK cells (Natural Killer cells) and B cells. Its interaction with PD-L1 on tumour cells suppresses cytotoxic T-cell responses and promotes immune evasion. Immune checkpoint inhibitors (ICIs) targeting PD-1 or PD-L1 restore T-cell activity and antitumour immunity.^57^

The rationale for ICIs in GCCs stems from their immune-rich tumour microenvironment, characterised by macrophages, lymphocytes, NK cells and dendritic cells.^58,59^ PD-L1 expressions are common, particularly in embryonal carcinoma and seminoma, but varies between studies. High PD-L1 expression in tumour cells has been associated with poor outcomes, whereas PD-L1 expression in tumour-infiltrating lymphocytes may correlate with improved survival.^60,61^

Despite frequent immune-cell infiltration and variable PD-L1 expression, most GCC exhibit a comparatively low tumour mutational burden and consequently a low neoantigen load. This biological feature likely constrains the generation of strong, mutation-driven tumour-specific T-cell responses and may contribute to the consistently low response rates observed with PD-1/PD-L1 blockade. Taken together, an immune-infiltrated microenvironment alone appears insufficient to predict benefit in refractory GCCs in the absence of higher neoantigenicity.

Early-phase clinical trials of ICIs in refractory GCCs have shown limited or no efficacy. In the phase II GU14-206 trial, pembrolizumab induced no objective responses in 12 non-seminoma patients.^62^ Small case series reported isolated durable remissions with PD-1 blockade, mostly in PD-L1-positive tumours.^63^ In a Japanese phase II study, nivolumab produced 1 PR and 3 SDs among 17 patients, with benefit unrelated to PD-L1 expression but linked to high tumour mutational burden.^64^ Another pembrolizumab trial achieved only SD in 3 of 12 cases.^65^

Anti-PD-L1 therapy with avelumab failed to produce responses in eight patients.^66^ In the APACHE study, durvalumab ± tremelimumab led to 1 PR and 1 SD among 22 patients (both patients were in combination arm), but hyperprogression occurred in up to 72% of cases.^67^

Overall, checkpoint inhibitors have not demonstrated clinical benefit in refractory GCCs. Responses, when observed, are rare and short-lived, and no validated predictive biomarkers currently support routine ICI use in this setting (Table 2).

**Table 2.** Immunotherapy trials with checkpoint-inhibitors in platinum-refractory GCTs.

Clinical trial/phase	Retrospective off-label case series	Drugs/targets	Patients	Clinical outcomes	References
GU14-206/phase II	/	Pemb/PD1	12 TGCT	Two radiographic SD for 28 and 19 weeks, respectively. No CR or PR. Six late relapses (>2 years).	(62)
/	Yes	Pemb or Nivo/PD1	7 mGCT	Three pts were treated for at least 6 months; two achieved long-term tumour response, both had high PD-L1 expression.	(63)
UMIN000028249/phase II	/	Nivo/PD1	17 mGCT	1 PR (mDOR: 90 weeks), 2 SD (mDOR: 68 weeks). PD-L1 expression not associated with ORs.	(64)
NCT02721732/phase II	/	Pemb/PD1	12 mGCT	no ORs, 3 pts had transient SD. mPFS of 2.4 mo; mOS 10.6 mo.	(65)
NCT03403777/phase II	/	Avel/PD-L1	8 mGCT	12-week PFS: 0%; mPFS: 0.9 mo; mOS: 2.7 mo.	(66)
APACHE trial – NCT03081923/phase II	/	Treme ± Durva/anti-CTLA-4 ± anti-PD-L1	22 mGCT	Combination arm (11 pts): 1 PR and 1 SD (which lasts 3 mo).	(67)

Avel, Avelumab; CR, complete response; Durv, Durvalumab; mDOR, median duration of response; mGCT, metastatic germ cell tumour; mo, months; mOS, median overall survival; mPFS, median progression free survival; Nivo, Nivolumab; ORs, objective responses; PD, programmed cell death protein; Pemb, Pembrolizumab; PR, partial response; pts, patients; SD, stable disease; TGCT, testicular germ cell tumour; Treme, tremelimumab.

## Emerging targeted therapies for platinum-resistant testicular GCTs: Antibody–drug conjugates and chimeric antigen receptor-T

### Targeting CD30

CD30 is a transmembrane glycoprotein of the TNF receptor family involved in tumour cell growth regulation. While uniformly expressed in Hodgkin lymphoma and anaplastic large cell lymphoma, CD30 is also variably detected in GCCs, particularly in embryonal carcinoma (≈93%–99%) and in about 20% of seminomas.^68^ CD30 expression in post-chemotherapy residual viable GCC tissue has been linked to inferior survival.^69^

Brentuximab vedotin, an antibody–drug conjugate (ADC) targeting CD30, has shown strong efficacy in lymphomas. In a phase II basket trial including five GCC patients, two achieved objective responses (one CR, one PR).^70^ However, two subsequent phase II trials with 24 and 18 chemo-refractory GCC patients, failed to demonstrate meaningful clinical benefit with only short-lived serum tumour markers declines and disease stabilisation in a few patients.^71,72^

Importantly, several targeted-therapy studies in refractory GCCs – including ADCs – were conducted in largely unselected populations without biomarker enrichment, which may have diluted potential efficacy signals. Biomarker-driven selection (e.g. confirmed target expression in viable tumour tissue) should be prioritised in future trial design.

### Claudin-6 as a therapeutic target

Claudin-6 (CLDN6) is an oncofetal tight-junction protein normally confined to embryonic tissues and largely absent in adult organs.^73^ In several cancers – particularly GCCs, ovarian, endometrial and certain lung tumours – CLDN6 is aberrantly overexpressed and contributes to epithelial proliferation and differentiation.^74^ Nearly, all GCCs display CLDN6 expression, making it an attractive therapeutic target.

Multiple CLDN6-directed modalities are under clinical evaluation, including monoclonal antibodies, chimeric antigen receptor-T (CAR-T) cells, bispecific T-cell engagers (BiTEs) and ADCs.^69^ The monoclonal antibody ASP1650 was the first tested but showed no objective responses in a phase II trial, with only transient disease stabilisation in 3 of 19 patients.^75^

A more promising approach uses CLDN6-CAR-T cells (BNT211-01) in combination with the mRNA vaccine CARVac, which enhances CAR-T persistence by transient CLDN6 expression in antigen-presenting cells. Among 13 refractory non-seminoma patients treated, an ORR of ~60% and disease control rate (DCR) of 85% were achieved.^76^ However, cytokine-release syndrome occurred in 65%, cytopenias in 59% and fatal adverse events in ~9%.^77^

In the BNT142-01 trial (NCT05262530), an mRNA-encoded anti-CLDN6/CD3 bispecific antibody (RiboMab02.1) induced a dose-dependent immune activation and a 58% DCR in 65 patients (10 GCCs) with manageable toxicity.^78^

Two CLDN6-targeting ADCs have entered phase I evaluation: DS-9606a, a pyrrolobenzodiazepine-based ADC that induced PRs in 2 of 7 GCC patients and ⩾90% tumour-marker reductions in most cases,^79^ but whose clinical development was recently discontinued; and TORL-1-23, an Monomethyl auristatin E (MMAE)-conjugated ADC that achieved overall response rates of 26%–42% and disease-control rates of 68%–89% in the total study population, including six patients with GCCs.^80^

Together, these results identify CLDN6 as a promising therapeutic target in refractory GCCs, though current data remain limited and confirmatory trials are awaited (see Table 3).

**Table 3.** Clinical trials investigating novel therapeutic targets, CD30 and CLDN6, enrolling patients with refractory GCTs.

Target	Clinical trial number	Phase	Patients	Type of drug	Drug name	Clinical outcome	Status	References
CD30	NCT01461538	II	5 mGCT	ADC	BV	1 pts CR, 1 pts PR	Completed	(70)
CD30	NCT02689219	II	18 mGCT	ADC	BV	6 SD; no PR or CR. 5 pts had transient >50% decline in baseline AFP/hCG	Terminated	(71)
CD30	NCT	II	24	ADC	BV		Completed	(72)
CLDN6	NCT03760081	II	19 mGCT	mAb	ASP1650	No confirmed radiographic responses	Completed	(75)
CLDN6	BNT211-01 (NCT04503278)	I	13 mGCT	CAR-T cells ± CARVac	/	ORR: ~60%; DCR: 85%	Recruiting	(76)
CLDN6	NCT05394675	I	10 mGCT	ADC	DS-9606a	2/7 pts: PR; 5/7 pts: ⩾90% marker reduction	Active, not recruiting	(79)
CLDN6	NCT05103683	I	6 mGCT	ADC	TORL-1-23	ORR: 26%–42%; DCR: 68%–89% (all population)	Recruiting	(80)
CLDN6 and CD3	BNT142-01 (NCT05262530)	I/II	65 pts (10 mGCT)	BiTE (mRNA-encoded anti-CLDN6/CD3 bispecific antibody)	RiboMab02.1	DCR: 58% (all population)	Active, not recruiting	(78)
CLDN6 and CD3	NCT06276491	I	/	BiTE	XmAb541	/	Recruiting	(81)
CLDN6 and CD3	NCT06515613	I	/	BiTE	CTIM-76	/	Recruiting	(82)
CLDN6 and CD3 and CD137	NCT05735366	I	/	TriTE	SAIL66	/	Active, not recruiting	(83)
CLDN6, GPC3, Mesothelin and AXL	NCT05410717	I	/	CAR-NK therapy	/	/	Recruiting	https://clinicaltrials.gov/study/NCT05410717 (84)

Notes (ClinicalTrials.gov):

-Completed: The study has ended normally, and participants are no longer being examined or treated.

-Terminated: The study has stopped early and will not start again. Participants are no longer being examined or treated.

-Active, not recruiting: The study is ongoing, and participants are receiving an intervention or being examined, but potential participants are not currently being recruited or enrolled.

ADC, antibody drug conjugates; AXL, AXL receptor tyrosine kinase; BiTE, bispecific T-cell engager; BV, Brentuximab vedotin; CAR-NK, chimeric antigen receptor-natural killer cells; CAR-T, chimeric antigen receptor-T; CARVac, CAR T cell-amplifying RNA vaccine; CD137, cluster of differentiation 137; CD3, cluster of differentiation 3; CLDN6, claudin-6; CR, complete response; DCR, disease control rate; GPC3, glypican-3; mAb, monoclonal antibody; mGCT, metastatic germ cell tumour; OR, objective response; ORR, objective response rate; PR, partial response; pts, patients; SD, stable disease; TriTE, trispecific T-cell engager.

### Other ADCs targeting refractory GCCs

Given the histology-agnostic approval of trastuzumab deruxtecan for HER2-positive solid tumours, assessing HER2 in GCCs may be relevant. Although no GCC patients were included in DESTINY trials, early IHC studies found HER2 overexpression in ~35% of samples, mainly in post-chemotherapy or mediastinal GCCs, but absent in primary testicular tumours.^85,86^ Thus, HER2 testing may be justified in selected late-relapses or mediastinal cases.

TROP-2, expressed in about half of GCCs – especially choriocarcinomas and yolk-sac tumours – represents another potential ADC target. The TROP-2-directed ADC datopotamab deruxtecan is under evaluation across tumour types in the TROPION-PanTumour03 trial, though GCCs are not yet included.^87^ In addition, emerging descriptive pathology data suggest that Nectin-4 expression may be enriched in specific histologic subtypes, particularly choriocarcinoma, supporting further exploration of Nectin-4-directed ADCs in biomarker-selected cohorts.^88^

## Future directions and translational priorities

The therapeutic plateau in multiply relapsed, platinum-refractory GCCs highlights the need for biomarker-driven strategies that are tightly linked to tumour biology. A first priority is systematic molecular profiling of viable tumour tissue at relapse, integrating histology (including somatic malignant transformation), immunophenotyping and genomics/epigenomics to identify actionable vulnerabilities and to avoid target-negative enrollment.

From a translational perspective, CLDN6 represents the most compelling near-term target, with early clinical activity across CAR-T, BiTEs and ADC platforms. Key next steps include defining optimal patient selection (e.g. CLDN6 expression thresholds in viable tumour, spatial heterogeneity), improving safety management (cytopenias, CRS, infectious risk) and establishing sequencing concepts with surgery and cytotoxic salvage regimens.

Beyond CLDN6, a rational roadmap should include (i) histology-enriched targeting approaches (e.g. subtype-associated surface antigens), (ii) epigenetic re-sensitisation concepts that leverage the distinctive methylation biology of GCCs and (iii) combination strategies designed to overcome the low neo antigenicity that likely limits checkpoint inhibitor efficacy.

Finally, future trials should incorporate modern design features appropriate for this ultra-rare setting: biomarker enrichment, centralised pathology review, harmonised definitions of platinum resistance, mandatory reporting of surgical resection status and relapse histology and endpoints that capture durable disease control (including post-chemotherapy complete resection rates and long-term NED status). International collaboration and prospective registries will be essential to generate sufficiently powered datasets and to accelerate validation of emerging targets.

## Conclusion

Platinum-refractory GCCs remain a therapeutic challenge. While conventional chemotherapy and high-dose regimens still define standard care, novel molecularly targeted and immune-based approaches – particularly CLDN6-directed CAR-T, bispecific and ADC therapies – show early promise. Broader prospective studies and biomarker-driven selection will be key to establishing their clinical role.

## Declarations

**Ethics approval and consent to participate:** Not applicable. This article is a narrative review and did not involve new studies with human participants or animals performed by any of the authors.

**Consent for publication:** Not applicable.

**Availability of data and materials:** Not applicable. No new datasets were generated or analysed in this study.

## References

[1] Park JS Kim J Elghiaty A , et al. Recent global trends in testicular cancer incidence and mortality. Medicine (Baltimore) 2018; 97(37): e12390. 10.1097/MD.0000000000012390 PMC6155960 30213007

[2] Gillessen S Sauve N Collette L , et al. Predicting outcomes in men with metastatic nonseminomatous germ cell tumors (NSGCT): results from the IGCCCG update consortium. J Clin Oncol 2021; 39(14): 1563–1574. 33822655 10.1200/JCO.20.03296 PMC8099402

[3] McHugh DJ Feldman DR . Conventional-dose versus high-dose chemotherapy for relapsed germ cell tumors. Adv Urol 2018; 2018: 7272541. 29736168 10.1155/2018/7272541 PMC5874975

[4] International Prognostic Factors Study Group; Lorch A Beyer J Bascoul-Mollevi C , et al. Prognostic factors in patients with metastatic germ cell tumors who experienced treatment failure with cisplatin-based first-line chemotherapy. J Clin Oncol 2010; 28(33): 4906–4911. 20956623 10.1200/JCO.2009.26.8128

[5] Oing C Seidel C Bokemeyer C . Therapeutic approaches for refractory germ cell cancer. Expert Rev Anticancer Ther 2018; 18(4): 389–397. 29516750 10.1080/14737140.2018.1450630

[6] Oing C Giannatempo P Honecker F , et al. Palliative treatment of germ cell cancer. Cancer Treat Rev 2018; 71: 102–107. 30415106 10.1016/j.ctrv.2018.10.007

[7] Adra N Abonour R Althouse SK , et al. High-dose chemotherapy and autologous peripheral-blood stem-cell transplantation for relapsed metastatic germ cell tumors: the Indiana University Experience. J Clin Oncol 2017; 35(10): 1096–1102. 27870561 10.1200/JCO.2016.69.5395 PMC5455354

[8] Seidel C Schaefers C Connolly EA , et al. Efficacy and safety of high-dose chemotherapy as the first or subsequent salvage treatment line in patients with relapsed or refractory germ cell cancer: an international multicentric analysis. ESMO Open 2024; 9(5): 103449. 38744098 10.1016/j.esmoop.2024.103449 PMC11108831

[9] Lorch A Neubauer A Hackenthal M , et al. High-dose chemotherapy (HDCT) as second-salvage treatment in patients with multiple relapsed or refractory germ-cell tumors. Ann Oncol 2010; 21(4): 820–825. 19822531 10.1093/annonc/mdp366

[10] Fossa SD Gilbert E Dores GM , et al. Noncancer causes of death in survivors of testicular cancer. J Natl Cancer Inst 2007; 99(7): 533–544. 17405998 10.1093/jnci/djk111

[11] Kollmannsberger C Rick O Derigs HG , et al. Activity of oxaliplatin in patients with relapsed or cisplatin-refractory germ cell cancer: a study of the German Testicular Cancer Study Group. J Clin Oncol 2002; 20(8): 2031–2037. 11956262 10.1200/JCO.2002.08.050

[12] Fizazi K Culine S Chen I . Oxaliplatin in non-seminomatous germ-cell tumors. Ann Oncol 2004; 15(8): 1295. 15277272 10.1093/annonc/mdh307

[13] Bokemeyer C Gerl A Schoffski P , et al. Gemcitabine in patients with relapsed or cisplatin-refractory testicular cancer. J Clin Oncol 1999; 17(2): 512–516. 10080593 10.1200/JCO.1999.17.2.512

[14] Einhorn LH Stender MJ Williams SD . Phase II trial of gemcitabine in refractory germ cell tumors. J Clin Oncol 1999; 17(2): 509–511. 10080592 10.1200/JCO.1999.17.2.509

[15] Bokemeyer C Schmoll HJ Natt F , et al. Preliminary results of a phase I/II trial of paclitaxel in patients with relapsed or cisplatin-refractory testicular cancer. J Cancer Res Clin Oncol 1994; 120(12): 754–757. 7798304 10.1007/BF01194278 PMC12200731

[16] Sandler AB Cristou A Fox S , et al. A phase II trial of paclitaxel in refractory germ cell tumors. Cancer 1998; 82(7): 1381–1386. 9529032 10.1002/(sici)1097-0142(19980401)82:7<1381::aid-cncr23>3.0.co;2-1

[17] Bokemeyer C Beyer J Metzner B , et al. Phase II study of paclitaxel in patients with relapsed or cisplatin-refractory testicular cancer. Ann Oncol 1996; 7(1): 31–34. 10.1093/oxfordjournals.annonc.a010473 9081388

[18] Nazario A Amato RJ Hutchinson L , et al. Paclitaxel in extensively pretreated nonseminomatous germ cell tumors. Urol Oncol 1995; 1(5): 184–187. 21224115 10.1016/1078-1439(95)00064-x

[19] Motzer RJ Bajorin DF Schwartz LH , et al. Phase II trial of paclitaxel shows antitumor activity in patients with previously treated germ cell tumors. J Clin Oncol 1994; 12(11): 2277–2283. 7525885 10.1200/JCO.1994.12.11.2277

[20] Miller JC Einhorn LH . Phase II study of daily oral etoposide in refractory germ cell tumors. Semin Oncol 1990; 17(1 Suppl. 2): 36–39. 2154858

[21] Maroto P Huddart R Garcia del Muro X , et al. Brief report: phase II multicenter study of temozolomide in patients with cisplatin-resistant germ cell tumors. Oncology 2011; 80(3–4): 219–222. 21734411 10.1159/000329041

[22] Kollmannsberger C Beyer J Liersch R , et al. Combination chemotherapy with gemcitabine plus oxaliplatin in patients with intensively pretreated or refractory germ cell cancer: a study of the German Testicular Cancer Study Group. J Clin Oncol 2004; 22(1): 108–114. 14701772 10.1200/JCO.2004.06.068

[23] Pectasides D Pectasides M Farmakis D , et al. Gemcitabine and oxaliplatin (GEMOX) in patients with cisplatin-refractory germ cell tumors: a phase II study. Ann Oncol 2004; 15(3): 493–497. 14998855 10.1093/annonc/mdh103

[24] De Giorgi U Rosti G Aieta M , et al. Phase II study of oxaliplatin and gemcitabine salvage chemotherapy in patients with cisplatin-refractory nonseminomatous germ cell tumor. Eur Urol 2006; 50(5): 1032–1038; discussion 8–9. 10.1016/j.eururo.2006.05.011 16757095

[25] Pectasides D Pectasides M Farmakis D , et al. Oxaliplatin and irinotecan plus granulocyte-colony stimulating factor as third-line treatment in relapsed or cisplatin-refractory germ-cell tumor patients: a phase II study. Eur Urol 2004; 46(2): 216–221. 15245816 10.1016/j.eururo.2004.03.001

[26] Bokemeyer C Oechsle K Honecker F , et al. Combination chemotherapy with gemcitabine, oxaliplatin, and paclitaxel in patients with cisplatin-refractory or multiply relapsed germ-cell tumors: a study of the German Testicular Cancer Study Group. Ann Oncol 2008; 19(3): 448–453. 18006893 10.1093/annonc/mdm526

[27] Sadeghi S Quinn DI Tsao-Wei DD , et al. Phase II study of gemcitabine, oxaliplatin, and paclitaxel (GOT) on a 2-weekly schedule in patients (pts) with refractory germ cell tumor (rGCT): final results. J Clin Oncol 2013; 31(15_suppl): 4531.

[28] Necchi A Nicolai N Mariani L , et al. Combination of paclitaxel, cisplatin, and gemcitabine (TPG) for multiple relapses or platinum-resistant germ cell tumors: long-term outcomes. Clin Genitourin Cancer 2014; 12(1): 63–69.e1. 10.1016/j.clgc.2013.07.005 24161525

[29] Raggi D Giannatempo P Miceli R , et al. Etoposide, methotrexate, and dactinomycin alternating with cyclophosphamide and vincristine (EMACO) for male patients with HCG-expressing, chemoresistant germ cell tumors. Am J Clin Oncol 2017; 40(1): 60–65. 25089532 10.1097/COC.0000000000000113

[30] Oing C Hentrich M Lorch A , et al. Treatment of refractory germ-cell tumours with single-agent cabazitaxel: a German Testicular Cancer Study Group case series. J Cancer Res Clin Oncol 2020; 146(2): 449–455. 31838576 10.1007/s00432-019-03071-2 PMC11804402

[31] Orszaghova Z Kalavska K Mego M , et al. Overcoming chemotherapy resistance in germ cell tumors. Biomedicines 2022; 10(5): 972. 35625709 10.3390/biomedicines10050972 PMC9139090

[32] Shamash J Powles T Mutsvangwa K , et al. A phase II study using a topoisomerase I-based approach in patients with multiply relapsed germ-cell tumours. Ann Oncol 2007; 18(5): 925–930. 17355956 10.1093/annonc/mdm002

[33] Oechsle K Kollmannsberger C Honecker F , et al. Long-term survival after treatment with gemcitabine and oxaliplatin with and without paclitaxel plus secondary surgery in patients with cisplatin-refractory and/or multiply relapsed germ cell tumors. Eur Urol 2011; 60(4): 850–855. 21704446 10.1016/j.eururo.2011.06.019

[34] Seidel C Oechsle K Lorch A , et al. Efficacy and safety of gemcitabine, oxaliplatin, and paclitaxel in cisplatin-refractory germ cell cancer in routine care – registry data from an outcomes research project of the German Testicular Cancer Study Group. Urol Oncol 2016; 34(4): 167.e21-8. 10.1016/j.urolonc.2015.11.007 26699830

[35] Oechsle K Honecker F Cheng T , et al. Preclinical and clinical activity of sunitinib in patients with cisplatin-refractory or multiply relapsed germ cell tumors: a Canadian Urologic Oncology Group/German Testicular Cancer Study Group cooperative study. Ann Oncol 2011; 22(12): 2654–2660. 21415240 10.1093/annonc/mdr026

[36] Necchi A Lo Vullo S Giannatempo P , et al. Pazopanib in advanced germ cell tumors after chemotherapy failure: results of the open-label, single-arm, phase 2 Pazotest trial. Ann Oncol 2017; 28(6): 1346–1351. 28383677 10.1093/annonc/mdx124

[37] Skoneczna IA Natorksa U Tacikowska M , et al. Sorafenib monotherapy in patients with inoperable/recurrent germ cell tumors (GCT) refractory to chemotherapy: phase II study. J Clin Oncol 2014; 32(Suppl_4): 367.24419110

[38] King J Dropcho S Beeler S , et al. A phase II trial evaluating the efficacy of cabozantinib in the treatment of patients with refractory germ-cell tumors (GCT). J Clin Oncol 2022; 40(6_suppl): TPS428.

[39] Einhorn LH Brames MJ Heinrich MC , et al. Phase II study of imatinib mesylate in chemotherapy refractory germ cell tumors expressing KIT. Am J Clin Oncol 2006; 29(1): 12–13. 16462496 10.1097/01.coc.0000195086.47548.ef

[40] Fenner M Oing C Dieing A , et al. Everolimus in patients with multiply relapsed or cisplatin refractory germ cell tumors: results of a phase II, single-arm, open-label multicenter trial (RADIT) of the German Testicular Cancer Study Group. J Cancer Res Clin Oncol 2019; 145(3): 717–723. 30232558 10.1007/s00432-018-2752-z PMC11810252

[41] Mego M Svetlovska D Miskovska V , et al. Phase II study of everolimus in refractory testicular germ cell tumors. Urol Oncol 2016; 34(3): 122.e17-22. 10.1016/j.urolonc.2015.10.010 26612480

[42] Piombino C Cortesi L . Insights into the possible molecular mechanisms of resistance to PARP inhibitors. Cancers (Basel) 2022; 14(11): 2804. 35681784 10.3390/cancers14112804 PMC9179506

[43] Caggiano C Cavallo F Giannattasio T , et al. Testicular germ cell tumors acquire cisplatin resistance by rebalancing the usage of DNA repair pathways. Cancers (Basel) 2021; 13(4): 787. 33668653 10.3390/cancers13040787 PMC7917736

[44] Schepisi G Urbini M Casadei C , et al. Olaparib as a rescue treatment in platinum-refractory germ-cell tumors: the IGG-02 phase II trial. ESMO Open 2025; 10(5): 105056. 40279883 10.1016/j.esmoop.2025.105056 PMC12245464

[45] Mego M Svetlovska D Reckova M , et al. Gemcitabine, carboplatin and veliparib in multiple relapsed/refractory germ cell tumours: the GCT-SK-004 phase II trial. Invest New Drugs 2021; 39(6): 1664–1670. 34052929 10.1007/s10637-021-01130-5

[46] Parola S Oing C Rescigno P , et al. PARP inhibitors in testicular germ cell tumors: what we know and what we are looking for. Front Genet 2024; 15: 1480417. 39678373 10.3389/fgene.2024.1480417 PMC11638157

[47] Ramamurthy C Nassar AH Abou Alaiwi S , et al. Prevalence of pathogenic germline cancer risk variants in testicular cancer patients: identifying high risk groups. Urol Oncol 2022; 40(3): 113.e9–113.e15. 10.1016/j.urolonc.2021.12.014 35022142

[48] Beyrouthy MJ Garner KM Hever MP , et al. High DNA methyltransferase 3B expression mediates 5-aza-deoxycytidine hypersensitivity in testicular germ cell tumors. Cancer Res 2009; 69(24): 9360–9366. 19951990 10.1158/0008-5472.CAN-09-1490 PMC2795063

[49] Koul S Houldsworth J Mansukhani MM , et al. Characteristic promoter hypermethylation signatures in male germ cell tumors. Mol Cancer 2002; 1: 8. 12495446 10.1186/1476-4598-1-8 PMC149411

[50] Koul S McKiernan JM Narayan G , et al. Role of promoter hypermethylation in cisplatin treatment response of male germ cell tumors. Mol Cancer 2004; 3: 16. 15149548 10.1186/1476-4598-3-16 PMC420487

[51] Martinelli C Lengert AVH Carcano FM , et al. MGMT and CALCA promoter methylation are associated with poor prognosis in testicular germ cell tumor patients. Oncotarget 2017; 8(31): 50608–50617. 28881587 10.18632/oncotarget.11167 PMC5584175

[52] Lobo J Constancio V Leite-Silva P , et al. Differential methylation EPIC analysis discloses cisplatin-resistance related hypermethylation and tumor-specific heterogeneity within matched primary and metastatic testicular germ cell tumor patient tissue samples. Clin Epigenetics 2021; 13(1): 70. 33823933 10.1186/s13148-021-01048-y PMC8025580

[53] Biswal BK Beyrouthy MJ Hever-Jardine MP , et al. Acute hypersensitivity of pluripotent testicular cancer-derived embryonal carcinoma to low-dose 5-aza deoxycytidine is associated with global DNA damage-associated p53 activation, anti-pluripotency and DNA demethylation. PLoS One 2012; 7(12): e53003. 10.1371/journal.pone.0053003 PMC3531428 23300844

[54] Albany C Hever-Jardine MP von Herrmann KM , et al. Refractory testicular germ cell tumors are highly sensitive to the second generation DNA methylation inhibitor guadecitabine. Oncotarget 2017; 8(2): 2949–2959. 27936464 10.18632/oncotarget.13811 PMC5356854

[55] Oing C Verem I Mansour WY , et al. 5-Azacitidine exerts prolonged pro-apoptotic effects and overcomes cisplatin-resistance in non-seminomatous germ cell tumor cells. Int J Mol Sci 2018; 20(1): 21. 30577584 10.3390/ijms20010021 PMC6337423

[56] Albany C Fazal Z Singh R , et al. A phase 1 study of combined guadecitabine and cisplatin in platinum refractory germ cell cancer. Cancer Med 2021; 10(1): 156–163. 33135391 10.1002/cam4.3583 PMC7826483

[57] Kalavska K Schmidtova S Chovanec M , et al. Immunotherapy in testicular germ cell tumors. Front Oncol 2020; 10: 573977. 33072608 10.3389/fonc.2020.573977 PMC7542989

[58] Fankhauser CD Curioni-Fontecedro A Allmann V , et al. Frequent PD-L1 expression in testicular germ cell tumors. Br J Cancer 2015; 113(3): 411–413. 26171934 10.1038/bjc.2015.244 PMC4522642

[59] Qu N Ogawa Y Kuramasu M , et al. Immunological microenvironment in the testis. Reprod Med Biol 2020; 19(1): 24–31. 31956282 10.1002/rmb2.12293 PMC6955586

[60] Chovanec M Cierna Z Miskovska V , et al. Prognostic role of programmed-death ligand 1 (PD-L1) expressing tumor infiltrating lymphocytes in testicular germ cell tumors. Oncotarget 2017; 8(13): 21794–21805. 28423520 10.18632/oncotarget.15585 PMC5400624

[61] Cierna Z Mego M Miskovska V , et al. Prognostic value of programmed-death-1 receptor (PD-1) and its ligand 1 (PD-L1) in testicular germ cell tumors. Ann Oncol 2016; 27(2): 300–305. 26598537 10.1093/annonc/mdv574 PMC4751222

[62] Adra N Einhorn LH Althouse SK , et al. Phase II trial of pembrolizumab in patients with platinum refractory germ-cell tumors: a Hoosier Cancer Research Network Study GU14-206. Ann Oncol 2018; 29(1): 209–214. 29045540 10.1093/annonc/mdx680

[63] Zschabitz S Lasitschka F Hadaschik B , et al. Response to anti-programmed cell death protein-1 antibodies in men treated for platinum refractory germ cell cancer relapsed after high-dose chemotherapy and stem cell transplantation. Eur J Cancer 2017; 76: 1–7. 28262583 10.1016/j.ejca.2017.01.033

[64] Kawahara T Kawai K Kojima T , et al. Phase II trial of nivolumab monotherapy and biomarker screening in patients with chemo-refractory germ cell tumors. Int J Urol 2022; 29(7): 741–747. 35462438 10.1111/iju.14885 PMC9545636

[65] Tsimberidou AM Vo HH Subbiah V , et al. Pembrolizumab in patients with advanced metastatic germ cell tumors. Oncologist 2021; 26(7): 558-e1098. 10.1002/onco.13682 PMC8265349 33491277

[66] Mego M Svetlovska D Chovanec M , et al. Phase II study of avelumab in multiple relapsed/refractory germ cell cancer. Invest New Drugs 2019; 37(4): 748–754. 31152292 10.1007/s10637-019-00805-4

[67] Necchi A Giannatempo P Raggi D , et al. An open-label randomized phase 2 study of durvalumab alone or in combination with tremelimumab in patients with advanced germ cell tumors (APACHE): results from the first planned interim analysis. Eur Urol 2019; 75(1): 201–203. 30243800 10.1016/j.eururo.2018.09.010

[68] Pallesen G Hamilton-Dutoit SJ . Ki-1 (CD30) antigen is regularly expressed by tumor cells of embryonal carcinoma. Am J Pathol 1988; 133(3): 446–450. 2849300 PMC1880812

[69] Richardson NH Adra N . Novel therapeutics in refractory germ cell tumors. Curr Opin Oncol 2025; 37(3): 267–273. 40065678 10.1097/CCO.0000000000001129

[70] Albany C Einhorn L Garbo L , et al. Treatment of CD30-expressing germ cell tumors and sex cord stromal tumors with brentuximab vedotin: identification and report of seven cases. Oncologist 2018; 23(3): 316–323. 29222199 10.1634/theoncologist.2017-0544 PMC5905693

[71] Ashkar R Feldman DR Adra N , et al. Phase II trial of brentuximab vedotin in relapsed/refractory germ cell tumors. Invest New Drugs 2021; 39(6): 1656–1663. 34031784 10.1007/s10637-021-01134-1 PMC9534326

[72] Necchi A Anichini A Raggi D , et al. Brentuximab vedotin in CD30-expressing germ cell tumors after chemotherapy failure. Clin Genitourin Cancer 2016; 14(4): 261–264.e4. 10.1016/j.clgc.2016.03.020 27105722

[73] Qu H Jin Q Quan C . CLDN6: from traditional barrier function to emerging roles in cancers. Int J Mol Sci 2021; 22(24): 13416. 34948213 10.3390/ijms222413416 PMC8705207

[74] Du H Yang X Fan J , et al. Claudin 6: therapeutic prospects for tumours, and mechanisms of expression and regulation (Review). Mol Med Rep 2021; 24(3): 677. 34296304 10.3892/mmr.2021.12316 PMC8335585

[75] Adra N Vaughn DJ Einhorn LH , et al. A phase II study assessing the safety and efficacy of ASP1650 in male patients with relapsed refractory germ cell tumors. Invest New Drugs 2022; 40(5): 1087–1094. 35759134 10.1007/s10637-022-01276-w PMC10207925

[76] Mackensen A Haanen J Koenecke C , et al. CLDN6-specific CAR-T cells plus amplifying RNA vaccine in relapsed or refractory solid tumors: the phase 1 BNT211-01 trial. Nat Med 2023; 29(11): 2844–2853. 37872225 10.1038/s41591-023-02612-0 PMC10667102

[77] Haanen JBAG Mackensen A Schultze-Florey C , et al. Updated results from BNT211-01 (NCT04503278), an ongoing, first-in-human, phase I study evaluating safety and efficacy of CLDN6 CAR T cells and a CLDN6-encoding mRNA vaccine in patients with relapsed/refractory CLDN6+ solid tumors. Ann Oncol 2024; 35: S489–S490.

[78] Yap TA Hernando-Calvo AH Calvo E , et al. First-in-human phase I/II trial evaluating BNT142, a first-in-class mRNA encoded, bispecific antibody targeting Claudin 6 (CLDN6) and CD3, in patients (pts) with CLDN6-positive advanced solid tumors. J Clin Oncol 2025; 43(16_Suppl): 2501.

[79] Patel MR Hamilton EP Piha-Paul S , et al. Preliminary results from a phase I, first-in-human study of DS-9606a, a claudin 6 (CLDN6)-directed antibody-drug conjugate (ADC), in patients with tumor types known to express CLDN6. Ann Oncol 2024; 35: S488–S489.

[80] Konecny GE Wahner Hendrickson AE Winterhoff B , et al. Phase I, two-part multicenter first-in-human (FIH) study of TORL-1-23: a novel claudin 6 (CLDN6) targeting antibody drug conjugate (ADC) in patients with advanced solid tumors. Ann Oncol 2024; 35: S551.

[81] Levitz D Gutierrez M Winer IS , et al. A phase 1, first-in-human, dose escalation and expansion study to evaluate the safety and tolerability of XmAb541 (claudin-6 x CD3) T-cell engaging bispecific antibody in subjects with germ cell tumors and other advanced solid tumors. J Clin Oncol 2025; 43(5_suppl): TPS652.

[82] O’Cearbhaill RE Barve MA Darus C , et al. A phase 1, first-in-human study of CTIM-76, a claudin-6 (CLDN6)-directed bispecific antibody, in patients with recurrent ovarian cancer and other advanced solid tumors. J Clin Oncol 2025; 43(16_suppl): TPS2685.

[83] Kamikawa T Kimura N Ishii S , et al. SAIL66, a next generation CLDN6-targeting T-cell engager, demonstrates potent antitumor efficacy through dual binding to CD3/CD137. J Immunother Cancer 2024; 12(10): e009563. 10.1136/jitc-2024-009563 PMC11474890 39401967

[84] Li J Hu H Lian H , et al. NK-92MI cells engineered with anti-claudin-6 chimeric antigen receptors in immunotherapy for ovarian cancer. Int J Biol Sci 2024; 20(5): 1578–1601. 38481806 10.7150/ijbs.88539 PMC10929190

[85] Pfister D Richter S Thuer D , et al. Her-2/neu expression in testicular cancer – a retrospective analysis in 57 cases. Urology 2010; 76(5): 1266.e6-9. 10.1016/j.urology.2010.06.036 21056276

[86] Soule S Baldridge L Kirkpatrick K , et al. HER-2/neu expression in germ cell tumours. J Clin Pathol 2002; 55(9): 656–658. 12194993 10.1136/jcp.55.9.656 PMC1769753

[87] Richardson N Salous T King J , et al. TROP-2 expression in germ cell tumors (GCT). J Clin Oncol 2025; 43(16_Suppl): 5031.

[88] Ricci C Ambrosi F Franceschini T , et al. Immunohistochemical expression of Nectin-4 and potential implications for enfortumab vedotin therapy in germ cell tumors of the testis: a preliminary study. Virchows Arch 2026; 488(3): 499–510. 40936013 10.1007/s00428-025-04243-x

